# The Efficacy of Family Protection Orders in Papua New Guinea: The Applicants’ Perspective

**DOI:** 10.1177/10778012251351912

**Published:** 2025-06-26

**Authors:** Judy Putt, Lindy Kanan

**Affiliations:** 1 Department of Pacific Affairs, 170491 Australian National University College of Arts and Social Sciences, Canberra, ACT, Australia

**Keywords:** domestic violence, protection orders, safety

## Abstract

Family protection orders were introduced into Papua New Guinea (PNG) in 2014. This paper reports on the findings from a study that examined the uptake, implementation, and efficacy of the orders, in a pluralistic and diverse country where domestic, family, and sexual violence is widespread. Adopting a safety-first and collaborative approach, the mixed methods study included more than 200 interviews with 118 order applicants in seven locations across PNG. The results were promising, with an increasing number of survivors applying for orders until the COVID-19 pandemic disrupted justice services. The paper argues that although based on an externally developed model of intervention from high-income countries, the protection order regime in PNG is worthy of further consolidation and expansion.

## Introduction

Globally, there was a rapid rollout and enactment of domestic violence legislation from the 1990s onwards in low-middle-income countries, from four countries in 1993 to 76 in 2013 (Ellsberg et al., 2015) and 162 countries in 2023 (UN Women, 2023). The law reforms focus on a criminal justice response as well as, in many countries, the introduction of civil protection orders. It is the latter which we focus on in the paper because, to date, there is very little evidence from low-income countries on how the civil orders have been implemented, whether they are accessed and if so, what are the outcomes. This is vital to know, as the civil protection regimes are modeled on interventions in high-income countries, and it is unclear how they will translate in a low-middle-income country, with high rates of domestic and family violence (DFV)^1^ and a pluralistic, resource-constrained, and gender-biased justice sector.

In this article, we report on findings from a research project on this very subject in the country of Papua New Guinea (PNG). As the next section outlines, PNG introduced family protection orders (FPOs) through legislation more than a decade ago as part of ongoing efforts to improve responses to common and entrenched DFV. Based on the existing literature of the impact of such reforms in high-income countries and their widespread adoption in low- and middle-income countries, the research sought to address the core question of how the regime had been implemented in a setting that is culturally diverse, politically turbulent and with impoverished government services. Given the critiques of civil protection orders, and of applying a western informed justice intervention, it was vital to document the manner in which the law operated in practice, before considering the fundamental issue of whether DFV survivors had benefited from the Act and the orders. From the research, which adopted a mixed methods approach, we focus on here the self-reported experiences of women who had applied for orders in seven locations across PNG. Their accounts, coupled with court and police data, and stakeholder perspectives, led us to conclude that the orders are worth pursuing but the process and system requires further reform and a significant investment by the government and donors in order to become accessible to more women (and men) in PNG who require protection.

## Context and Literature Review

### Papua New Guinea Context

Situated on the rim of the Pacific Ocean, adjacent to Australia and Indonesia, PNG is the most populous of the Melanesian countries with more than 10 million people. In a country renowned for its social diversity and fragmented geography, the state is viewed as fragile and one that struggles to provide essential services to the country's widely dispersed and fast-growing population. PNG is classified internationally as a low-income country, high on the corruption perceptions index and low on human development and gender inequality rankings (Global Women's Institute, 2022). Exploitation of the country's rich endowment of natural resources has delivered few tangible benefits to most citizens and, on the contrary, has been seen by some observers as exemplifying a resource curse that has accentuated corruption, uneven development, intense contestation over benefits distribution, as well as secessionist pressures in some areas (Dinnen, 2001). Rapid urbanization and population growth add to existing pressures and demands on government services, while the ongoing COVID-19 pandemic has had devastating social and economic impacts across the country (Mansour, 2021). The country is dominated by the Christian faith (96%) and is conservative in many respects, for example, same-sex relationships are illegal.

### PNG Legal Context and Family Protection Orders

PNG is referred to as a nation-state with a pluralistic or hybrid law and justice arena, with a confluence of legacies related to the traditional and colonial past, the history of missionization and the churches’ provision of services, and state reforms introduced just prior to and post its 1975 independence (Dinnen, 2001; Jolly, 2012). In terms of structuring state responses to gendered violence, legal, and policy reforms show the influence of external and western-oriented approaches to address the violence, demonstrated by the practical support of donor agencies and their emphasis on improving gender equity and reducing the subordinate status of women.

A critical reform in PNG was the introduction of the Family Protection Act (2013) (FPA), which introduced the specific criminal offense of domestic violence and a civil regime of FPOs. The PNG FPA largely uses language and concepts that echo laws in high-income western countries, although there is some recognition of local or traditional values and processes (Putt et al., 2019). The key institution that was assigned the responsibility of issuing interim and longer orders was the district court. In PNG's formal justice system that dates from the days of the Australian administration, the district courts sit at mid-level in the hierarchy of courts, and deal with primarily non-indictable criminal offenses and civil laws, notably family law (Kanan & Putt, 2021). Based in the urban centers of PNG's 24 provinces, the district courts have a high volume of cases, including domestic violence matters (Ganai, 2017).

While in many respects the Act represents an orthodox framework that creates the avenue of protection orders for specified people in specified situations, the Act and the subsequent guidelines (Department of Justice and Attorney General, 2017) do include aspects that recognize distinct features of the PNG context. To make the orders accessible, especially in rural areas, these include making it clear the process is free, and empowering the village courts, which are the country's most accessible courts, to issue interim protection orders (IPOs). In addition, the district court can order counseling, and at the time of issuing a protection order can order the respondent to pay compensation in accordance with custom.

### Protection Orders in the Global Literature

Introduced in the 1980s, the civil regimes of protection or restraining orders have become a mainstay in the statutory response to DFV, which complements the focus on domestic violence as a crime, in high-income countries. In theory, the advantages of the civil orders are that they can be timely, require a lower standard of proof than a crime, and give the survivor the option of applying for one and to seek conditions that are tailored to the circumstances (Chan, 2017; DeJong & Burgess-Proctor, 2006; Richards et al., 2018). Studies have highlighted that there is no equity in access and that the orders do not always offer protection, and can in some instances create more risk for the survivor (Capshew & McNeece, 2000; Conner, 2015). By and large though, in high-income countries, the evidence is that they are a useful option, that in many instances have helped the survivor and improved the safety of applicants and their dependents (Cordier et al., 2021; Dowling et al., 2018).

Feminist critiques of civil protection or restraining orders have included concerns about diluting the focus on criminal responses to DFV and the obscuring of the fundamental gendered hierarchies within the justice system and in society (Goodmark, 2018; Messing et al., 2021). More pertinently for low-middle-income countries, the global push for their introduction through state laws has been justified on the grounds of fundamental human rights and the legal empowerment of female citizens (Bott et al., 2005). However, in non-western and less affluent settings, law reform occurs in plural or hybrid justice-scapes, with vernacularization of formal legal concepts and justice processes and in an environment that is often precarious with resource-poor state institutions of limited reach, especially outside of urban centers (Levitt & Engle Merry, 2009). It is for these reasons that it is important to distinguish between what works to prevent domestic violence in high-income countries, compared with low-middle-income countries (Colucci & Hassan, 2014).

Encouraged by UN agencies, aid donors, and civil society organizations, legislation that establishes a civil regime of restraining or protection orders has been introduced in many other parts of the world in the previous few decades, typically embedded in domestic violence legislation that also underlines the criminality of the behavior. UN Women (2023) estimates that now more than 162 countries have passed laws on domestic violence although their website cautions against assuming that they are implemented or enforced. Only a limited literature exists on how well these regimes have been implemented, and whether they suit low- and middle-income countries and diverse contexts (Global Women's Institute, 2022; Heise, 2011). Drawing on the seminal work of Engle Merry (2006) several ethnographic studies have highlighted the vernacularisation of the law that occurs during the implementation of domestic violence laws, and local resistance to reforms to address gender-based violence (Goodale, 2017).

Emerging literature from low-middle-income countries shows that implementation of civil protection order regimes can be challenging, not only because of limited resources but also a range of other factors. Research in Mexico found that the protection offered by protection orders is limited because of insufficient institutional resources and prevailing attitudes towards gender-based violence (Frías, 2024). In Türkiye there is a culture of silence around domestic violence and protection orders are mostly used when the level of violence and abuse becomes unbearable (Turhan et al., 2025). Turhan et al. (2025) also found that protection orders are less likely to be effective if the victim-survivor lacks financial stability and social support. In Uruguay, Gambetta and Vanoli-Imperiale (2025) argue that it is the police response and data collection that needs improvement and investment to reduce revictimization of protection order holders. India has experienced difficulty in implementation of protection orders because of, inter alia, insufficient numbers of appointed “Protection Officers” relative to the population size, roles that are vital to the success of the legislation (Chan, 2017).

### Protection Orders in the Pacific Region

In the Pacific region, 2008–2018 marked an intense decade of domestic violence law reform, and protection order frameworks were introduced in 14 countries, including PNG (Maravuakula, 2022). A regional symposium on FPOs, involving participants from 10 countries across the South Pacific region revealed that although similar laws had been introduced in their jurisdictions, it was implementation of those laws that presented the most acute challenges. These challenges related to local values (domestic violence is dealt with by or within families); local processes extrinsic to the formally established justice process (mediation and compensation); limited capacity, ineptitude, and resistance within key justice agencies, notably the police (Putt & Kanan, 2021). Cokanasiga (2023) laments that the implementation of protection order systems in the Pacific has mainly been underpinned by feminist and human rights approaches without any consideration of the colonial experiences of Indigenous people. The often significant role of community leaders and chiefs, who draw upon customary authority, in diluting the intention of domestic violence laws has been described in Vanuatu (Tyedmers, 2024) and in the Solomon Islands (Ride & Soaki, 2019).

## Rationale for the Research

In addition to the question of *suitability* of an imported western model of legal intervention into a low- or middle-income country with pluralistic law and justice context that draws on colonial, traditional, and missionization roots, there was the question of how *feasible* it was to implement a civil regime in a country that has a limited state presence outside of urban areas and decentralized and under-resourced government services (Wiltshire, 2016). A dire picture is drawn by a Special Parliamentary Committee in PNG^2^ of the formal justice sector, of widespread reliance on informal justice processes to manage disputes and offenses, and uneven access to victim support services through non-government organizations (NGOs) and faith-based organizations (FBOs). Importantly, research indicates high levels of intimate partner violence in PNG, widespread values and practices that normalize domestic violence or make it the fault of the woman, structural barriers that constrain women which are linked to their subordinate political-economic status, and a failure on the part of the formal and informal avenues in the system to enable women to adequately access protection and justice (Craig & Porter, 2018; Global Women's Institute, 2022; Office of Development Effectiveness, 2019).

The research that we report on here aimed to capture both the vernacular translation of the Act on the ground, in relation to the way procedures and practices were implemented, but also how local actors judged the effect of the FPOs introduced under the Act on what might be termed justice and safety outcomes for the survivors of DFV. Central to this task is the victims’ own sense of justice and safety within the local contexts in which they live, which for many, includes being enmeshed in dense and sometimes onerous social relationships with family and others (called *wantoks* in PNG). In this paper, we present findings related to key research questions that focused on implementation of the FPA and on women's safety. The project investigated the use and efficacy of the civil protection orders by seeking data on how accessible the orders are, whether they are being issued, and whether they result in positive outcomes for the victims of DFV. There is very little academic literature specifically relating to FPOs in PNG, if any at all, which is why this paper fills an important gap.

## Methods

A pilot study, followed by a more extensive study, was conducted over several years and involved a research team of more than 25 people comprising a network of local researchers and specialist family and sexual violence services, and a number of PNG and Australian academics.

Working with PNG researchers and specialist services was essential given the potential safety risks associated with research on DFV (Ellsberg & Heise, 2005), as well as the language skills and deep cultural knowledge that local researchers bring. However, it does mean that we were more likely to interview clients of these services; more is said on this below.^3^ More detail on the research team, funding, methodology, and sites of the research has been documented in reports on the pilot and main studies, with the latter generating a series of papers and short videos (for project outputs, see Putt & Kanan, 2021). A submission was also made to the PNG Special Parliamentary Committee on gender-based violence. A range of methods were employed during the project, including a survey of 180 young adults, research team members conducted observations in district courts in the two main urban centers, meetings, and interviews were conducted with over 140 stakeholders, and justice and client statistics were reviewed and collated where available.

In addition, 211 interviews were conducted with 118 interim protection order applicants across 7 locations. The interviewees were almost all women (96.6%), mainly middle-aged (with an average age of 35.2 years), and more than half lived in town or an urban settlement, and the rest were primarily residents in villages. The majority (89.7%) had children living with them at home, with an average of 2.8 children per participant. A significant minority (31.6%) said they had poor English literacy (in reading and writing). The study involved two samples of IPO applicants. Originally, we had planned to conduct follow-up interviews at three time intervals after the initial interview with applicants, but due to a low volume of applicants in several sites, the difficulties in following up over time, and the state of emergency declared in response to COVID-19, we had to amend our approach mid-way through. Instead, we interviewed those who had applied for an IPO in the previous year. As a result, two samples were interviewed—Sample A (*n* = 61) had applied for an IPO in the previous year, and Sample B (*n* = 57 at first interview, and declined to *n* = 23 at fourth interview) were first interviewed around the time of their IPO application.

The study received approval from the Australian National University Ethics Committee, and the protocol emphasized a safety-first and collaborative approach. Locally based researchers with knowledge of DFV and the local context, and with pre-existing contacts with networks of women, played an essential role in approaching participants, carefully explaining the research, conducting the interviews, and following up over time with participants. They too assisted in the interpretation of the results from the interviews. Structured questionnaires were used for the interviews, which allowed for quantitative measures for the main topics covered, such as socio-demographic characteristics of participants, their experiences of the process, and their perceptions of the orders’ impact over time. Further thematic analysis was undertaken of open-text responses, with the local researchers assisting the compilation and prioritization of themes.

## Findings

This article presents the findings of the study organized around two key areas—implementation and efficacy. Firstly, the implementation section answers research questions including “how many orders are being granted” and “how is the process being conducted”? Next, the efficacy section deals with the research question on “are the orders meeting the needs of complainants” and covers the impacts that the orders are having on the applicants and respondents, as well as the impacts of counseling conducted in conjunction with the orders, and the impact of orders on couple's continued co-residence. The last section presents the findings relating to negative consequences of orders and ongoing violence.

### Implementation of Family Protection Orders

#### Uptake and Variability

**Figure 1.**
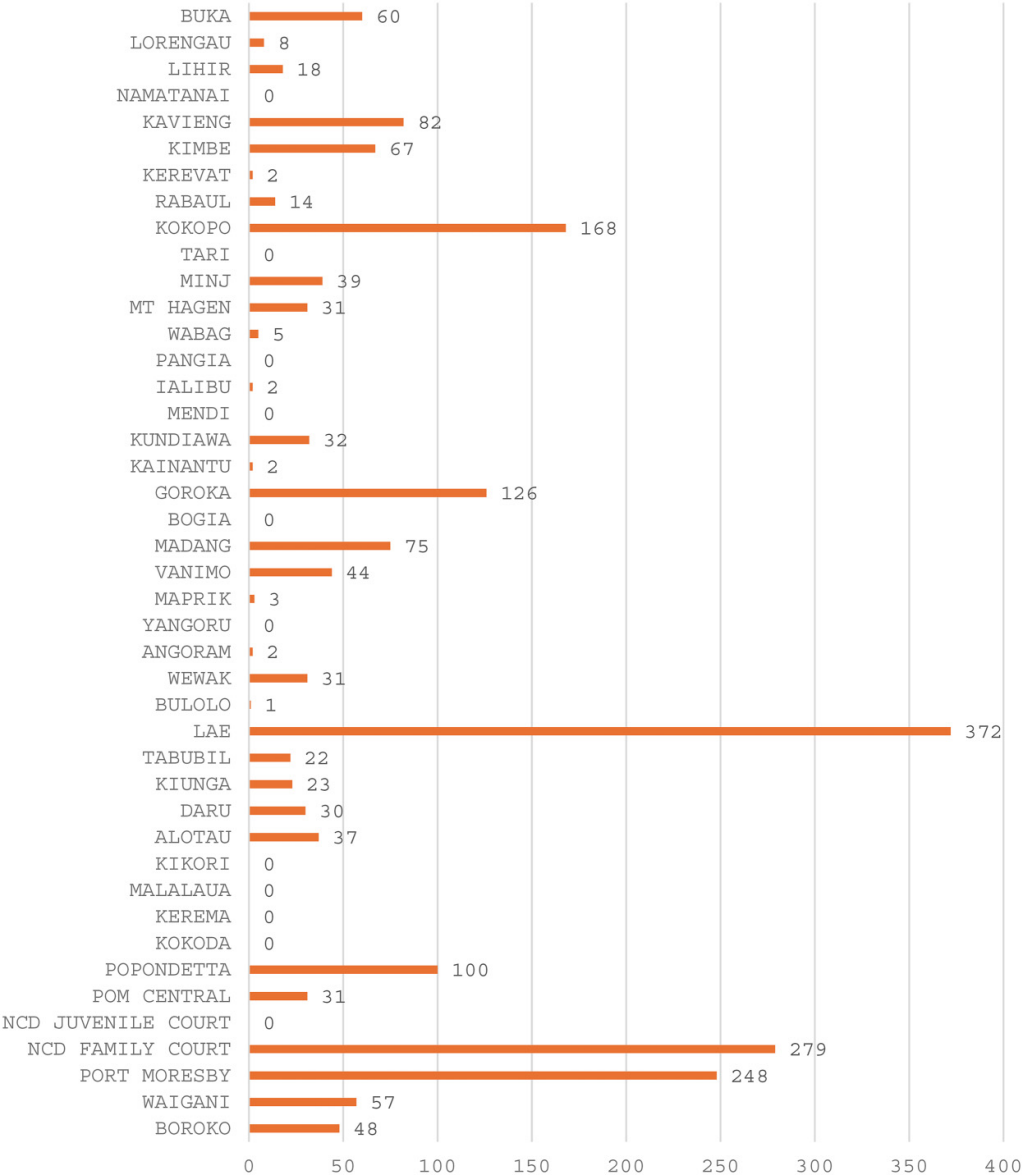
Number of IPO Applications Registered by District Courts, 2018.

*Source.* Magisterial Services, PNG.

Under the FPA, there are two types of orders—an IPO and a PO, with the IPO usually issued for a period of 30 days. The PO is a longer term order of up to 2 years in duration; an IPO can be converted to a PO or a PO can be issued as a standalone order. Applications are lodged with a clerk in the district courts, and it is the court magistrate that issues the orders and sets conditions if one is granted. Data we obtained from magisterial services for 2018 showed a wide range in the number of orders being applied for in the district courts, which are almost all located in the urban provincial capitals (see Figure 1). To date, we have been unable to secure more up-to-date statistics, but a number of courts that shared their statistics with us, and NGO service data, showed that the numbers in general were increasing up until COVID-19 disrupted services in 2020, and that unless the applicant was a client of a specialist FSV service, very few IPOs were converted to POs. Crime and safety surveys conducted in 2015, 2018, and 2022 indicate awareness of IPOs was growing among the general population (Sustineo, 2023), but our research showed that not many people, not even applicants and stakeholders, were aware of the availability of the longer term POs and the need to either separately apply for one or to have an IPO converted to one.

As well as variability in numbers across courts, there was a considerable variation in time it took to get an order, with some courts issuing IPOs relatively quickly. Other courts were more tardy, with often multiple adjournments as the sitting magistrates believed it was important to give the respondent an opportunity to present his (typically) perspective, although this is not a requirement under the Act.^4^ Another area of implementation where we found variability was in whether counseling was ordered, although it was not often and only by a few district court magistrates. Based on our court observations, it was the engaged and committed magistrates who were more likely to take on a counseling or mediation role themselves and they typically did this by inviting a couple into the privacy of his or her chambers where they would speak with the couple for some time, and several ordered counseling by church organizations and deferred a hearing to allow this to happen.

Justice stakeholders and IPO applicants who were interviewed stressed how frustratingly slow the formal justice system could be. For those applicants who were successful in obtaining an IPO, on average it took 14.8 days, but in some sites, it could be months before a decision was reached or the case was withdrawn or struck out. For instance, when asked what would have made them feel safer, based on 45 IPO applicants in Sample A^5^ who provided open-ended responses, almost half (43%) indicated improvements to services and justice responses were viewed as important. In both samples, police action^6^ was singled out as especially important along with, in Sample B, who were going through the process of applying for an order, somewhere to live, and the support of family and friends.

We believe a number of interrelated factors help explain the variability in numbers and poor uptake in some areas. Most obvious were the impoverished and frayed formal justice system, which is under-resourced and personnel frequently lacking in contemporary knowledge of reforms. In addition, there is a bureaucratic culture that fosters inertia and a reliance on paperwork,^7^ and the resultant costs (in money and time) are borne by the applicant. For example, applicants bear transport costs and sometimes have to serve documents themselves, even if the regulations make it clear that applications are free and that police can serve summons and IPOs.

While discretion is found in all aspects of the formal justice sector in high-income countries, notably in frontline services (Ohlin & Remington, 1993) and the policing response to domestic violence (Myhill & Johnson, 2016), the degree to which justice stakeholders in PNG can exercise discretion is amplified in a context where there is only tenuous accountability, for funds that are allocated centrally, and of staff at the local level with very little oversight from the central institution. Therefore, it is unsurprising that there is a high degree of latitude to interpret the Act in its implementation by magistrates and other local justice stakeholders. An example was where several frontline police were of the view that an IPO and criminal charges could not be pursued concurrently, or that other charges could not be laid concurrently with an IPO or PO breach offense. Compounding the distinct views on how the family protection order regime should operate were assumptions about the intent of the Act and who was covered by the regime. The Act is gender neutral and defines domestic violence to include assaults, stalking and threatening and other behavior, and the kind of relationships that are covered are (opposite sex) spouses, ex-spouses, de-facto relationships, and family members, including those who are treated by the spouse as a family member). Same-sex relationships remain illegal in PNG and hence are not covered by the FPA.

Yet we were told by many people, including justice practitioners and professionals, that the Act was ‘only for women’ and that it “caused the break up of marriages.” In relation to the latter, in fact, the Act's stated intent is five-fold—to promote *safe, stable, and strong families*; as well as prevent and deter domestic violence at all levels of society; recognize that domestic violence of any kind is not an acceptable behavior; ensure that there is effective legal protection for the victims of domestic violence; and provide for punishment of persons who commit acts of domestic violence or who breach protection orders.

#### Urban and Rural Divide

A final issue concerning access to protection orders is the urban-centric nature of the formal justice system. In recognition that district courts were not accessible to many in rural and remote areas, the Act does contain the provision that village courts had the power to issue IPOs, although POs have to be obtained from the district courts. Established by legislation in 1973, just before independence was declared in 1975, and operating in both rural and urban areas, the village courts are a more hybrid form of court, with jurisdiction over disputes including more minor offenses and a range of land and other issues (Demian, 2023; Goddard, 2009). Local community leaders are appointed as magistrates, and the tenor of deliberation and outcomes aligned with traditionally and community-rooted justice processes, with the Village Court Act stating the overall aim of the court is to restore relations and achieve harmony.

Our research found that aside from the challenges of making magistrates and officials in more than 1,600 village courts gazetted in the country aware of the legislation, there was reluctance on the part of some village court magistrates in urban areas to issue IPOs. As one said, “I can issue a preventive order, and it has more teeth.”^8^ It is impossible to know how many IPOs have been issued by the village courts, but we suspect it is not many despite some training on the FPA undertaken with village court officials in several provinces. In effect, a design flaw exists in that there is no incentive for the village court magistrates to use IPOs when they can issue a preventive order for which they have control over and can impose penalties if it is breached, and that if a longer order, a PO, is deemed necessary and appropriate, under the FPA, this still involves going to a district court.

Out of our seven key conclusions,^9^ based on our study, three summarized and addressed these challenges in implementation. These were the system needing more resources and funding, improving police effectiveness, and increasing access to district and village courts and to having committed and informed magistrates. Factors that made it more likely that orders were being issued and increased the likelihood of them being effective, were the support of the church and family, and the local presence of a specialist FSV service^10^ and what we term a network of justice actors that create *localized islands of hope* which draw on the indigenous social ethos of mutuality. Whether to work on reducing impediments or to make substantive changes to the law and its regulations involves considering whether the effort is worth it. Such considerations are informed by what the applicants for orders said about their ease of access and, if they were issued, their efficacy.

### The Efficacy of Orders

The 211 interviews with 118 IPO applicants formed the basis of findings about the efficacy of the orders. Another potential measure of their efficacy—whether orders were breached—was not a robust indicator, as there were very few examples of such charges being laid (which appeared to be due to police inaction and low levels of reporting rather than an absence of breaches).

#### Impact on Applicant (and Children)

Of the total who were interviewed, the majority (81.3%, *n* = 107) were successful in obtaining an IPO. Of those who did, the majority (79.8%) said they felt safer. There was a similar distribution of responses across both samples, except that a greater proportion in Sample B reported feeling less safe. As other research has demonstrated, victims’ perceptions of safety and justice and responses to questions about the process and their current state of well-being can alter over time depending on circumstances and their experiences of the system (e.g., Holder, 2018). With just over 20 IPO applicants in Sample B who completed all four interviews, it was notable that their perceptions of safety became more polarized at the third and fourth interviews. The proportion who felt very unsafe after 1 month, 8.3%, increased to 13% after 2 months, while those feeling very safe increased from 54.2% to 65.2%. From the responses to open-ended questions, it was apparent that applicants felt it took time to determine whether the IPO would continue to have a positive effect on the respondent. It also seemed that many interviewees did not know that the order only lasted for 30 days and had expired.

The majority of interviewees who felt safer because of IPOs had positive comments about their impact on themselves and their family life. Some underlined how the violence had stopped, others stressed how there was respect shown to them, or how their mental health had improved. Comments often related to feeling empowered or more in control, including the following: “Improved my family life and full authority over my house while he lives with his new wife. I am now empowered and felt safe knowing that the orders are already in place.” Given how many of the IPO applicants had children, and the number of children, most aged under 18, it is understandable why the family as a whole was viewed as benefiting from an end to abuse and violence. In a society, where the emphasis is on relationality and the well-being of various social collectivities (such as the extended family, and the clan) it was apparent that the individual benefits of having an order were interconnected to the wider impact on family and community life.

The overwhelming majority of interviewees said they would suggest an IPO to a friend or family member—100% of Sample A and 93.6% of Sample B. This suggests that even those who did not obtain an IPO or found that it had no effect still saw it as worthwhile to apply for one and that, for most, applying for an IPO resulted in sufficient improvements in their own and their family lives to warrant advocacy. Despite the applicants’ experiences of the process and its outcomes varying considerably, several key factors emerged as affecting a person's perceptions of their safety including whether the couple separated, whether they reconciled, the applicants’ support (from family, relatives, and specialist FSV services), and the reactions to and compliance of the respondent to the order.

#### Impact on Respondent

The above findings indicate a measure of effectiveness for a short-term order, when one is issued. The question as what contributes to its effectiveness is best answered, according to our results, by the respondents’ response to knowing that one has been applied for and importantly, having to appear in court. Almost nine out of 10 respondents to the IPO applications were a partner or former partner (89.8% for Sample A and 86% for Sample B). How the respondent reacted to the issuing of the IPO was critical to whether the applicant felt safer. Even where a respondent had reacted in anger upon receiving a summons or the IPO, there was a high level of compliance with the order reported by the interviews. A total of 70.1% of 87 interviewees said the respondent had complied with the order, with similar proportions in both samples. For the majority, the issuing and serving of an IPO, and the fact that it was in a formal court and done by a magistrate, had a deterrent effect. Many respondents were reported to have complied with the order for three key reasons: fear of and respect for the law and the courts, an understanding or acceptance that their behavior needed to change and a range of practical reasons. The most common explanation for compliance was fear, which resulted in the respondent desisting and/or leaving the home, as this response illustrates: “Order was on paper and he was scared. And now he never touches me.”

Other research participants described the husband as being “humbled,” “defeated,” and “humiliated.” Several interviewees also referred to practical motives for compliance, such as wanting to see children or not being able to see the interviewee because she/he was staying with family. With Sample B, in response to a question about what process made a difference during 2 months after their IPO application, the most positive comments referred to the respondent appearing before the district or family court^11^ magistrate and/or the police being involved, as the following illustrates: “Appearing in court and listening to the magistrate give him his orders had a big impact on his behaviour afterwards and even his arrest and going to jail is a big relief for me.” Another respondent explained: “Seeing police assisting me and how the magistrate made him see how serious his actions are made him change his behaviour.”

#### Impact of Counseling on Couples

For the small number of interviewees who reported being involved in counseling, as part of an IPO, they said the experience had a positive and educative effect on the respondent and/or the couple. Some interviewees indicated that their partner/ex-partner realized they had acted wrongly, typically as a result of counseling. Six interviewees viewed mediation as helpful. The interviewees appeared to be referring to either court-arranged counseling or to where the magistrate mediated between both parties about their differences, as was the case in one location. One interviewee said that “After mediation, we both listened to each other and he stopped womanising. Always at home with us and attending to family duties.”

Given that so few successful applicants had experienced counseling, or what some termed mediation, it is it is unclear whether the limited uptake of the provision in the FPA legislation is due to lack of knowledge, resistance to the notion, and/or limited accredited counselors and services available in PNG. More specific research is required to better understand what is understood as counseling, the different modes of delivery, and why it appears to result in positive outcomes where it is accessed.

#### Impact of an Order on a Couple's Continued Co-Residence

Based on previous research in PNG that revealed economic barriers for women (Human Rights Watch, 2015) and common perceptions that protection orders cause separation and favor women), we did ask applicants about their financial means of support, marital status and places of residence at the time of interview and after an IPO was issued. Of 118 IPO applicants only 16.1% said they had no income or that the source of their income was their husband or relatives. The majority of interviewees in both Samples A and B had an income from either a salary/wages (28%) or from gardening and/or marketing^12^ (54.2%), although the latter group was finding that the state of emergency restrictions were creating uncertainty and hardship. As a result, the majority of Sample A (74.6%) said they had the financial means to live on their own, but a slightly lower proportion (61.4%) of Sample B said the same. Importantly, for both samples, three-quarters said they did have the support of family, 71.2% (*n* = 59) and 75.4% (*n* = 57), respectively.

Interpreting these findings is aided by nuanced and in-depth appreciation of contemporary gender and spousal roles, and women's different degrees of autonomy and agency that flow from income generated by paid work, selling and trading, and growing food, and their connectedness to complex kin and place networks, despite male domination in the public, political and economic spheres (Jolly et al., 2015). For many respondents, with self-reported independent financial means and the support of her family, separation from the partner/husband is an option. The results from the study indicate that quite a few women move out of home at the time of crisis, often staying in a safe house run by an NGO or church, and then apply for an IPO as a response to the crisis. Some of them, depending on how the respondent behaved and other factors, then return home. Several interviewees commented that they would have preferred to return to their province or place of origin. We also found that an order can be in place but the couple continues to cohabitate. In short, there is very little evidence that applying for an IPO or having one issued triggers long-term separation. Several district court magistrates said they go out of their way to explain to respondents that even where the applicant and respondent are living apart and the order^13^ confirms that this should continue, it is only a short-term measure.

#### Negative Consequences and Ongoing Violence

In a number of different scenarios, perpetrators continue to commit DFV, and their behavior may even escalate. These scenarios include situations where DFV continues despite an IPO being issued and situations where the applicant was not successful in obtaining an IPO. Based on interviews with IPO applicants, some respondents did react badly when they found out an order had been applied for and/or issued. A minority were angry (16.9%) and a lower proportion were reported to have reacted furiously and/or were threatening (11.9%). A minority of interviewees (18.7%) did not obtain an IPO, which could relate to problems with the system and/or the applicant not wishing to pursue the order. A small but important number of applicants indicated that they continued to experience abuse and violence.^14^ However, while acknowledging that not all interviewees’ situations improved and some said they were worse off, our research suggests that the majority of applicants who obtain an IPO experience a cessation or reduction in abuse and violence, at least in the short term. More in-depth research is required to examine why the minority of respondents continue to act violently and are not deterred by the imposition of an order.

## Discussion

The implementation challenges associated with domestic violence legislation in Trinidad and Tobago, based on the work of Lazarus-Black (2001), accord with what we found. Echoing much of what was documented through our study, she refers to the public's limited knowledge of the legislation, the number of complainants not appearing in court, the variability in magistrates’ attitudes and practices, the difficulties of serving summons, men's fear of the police and going to jail, and only a few order breaches being recorded by police and/or reaching court. Like Lazarus-Black (2001) we identified factors that affect whether an order will be issued that relate to the circumstances of the applicant and those that are a product of a sector that is frayed, overburdened, and heavily dependent on the stance taken to DFV of critical actors within the sector.

The symposium on FPOs in the Pacific region also revealed a high level of congruity across Pacific nations in the issues that acted as barriers to access and protection (Putt & Kanan, 2022). This suggests a degree of commonality in the act of translating and making them work in low-middle-income countries, irrespective of the specific country context. From our research there was evidence that three of the FPA's five objectives were being met—to prevent and deter domestic violence at all levels of society; to recognize that domestic violence of any kind is not an acceptable behavior; and to ensure that there is effective legal protection for the victims of domestic violence—albeit for a subset of IPO applicants, and for a fraction of the adult female population in PNG. Having legislation and a formal justice system that is applying that law did communicate that DFV was not an acceptable behavior.

Our research findings also support what Lazarus-Black argues was a critical indicator of the law's success—*the symbolic value of state law, and that the women felt empowered* by its introduction, which gave them the opportunity to apply for an order (and typically it was often their first time in court), irrespective of the outcome. In an early article, Lazarus-Black (2001) framed her findings in terms of how the law resulted in limited access for only certain kinds of applicants and its symbolic significance, within the structural constraints and fluid negotiations that she calls “the pragmatics of inclusion” (which relates to agency and structural constraints, and ideological influences in reconstituting boundaries of appropriate/inappropriate familial and gender relationships). This is somewhat less than what might be claimed from a legal empowerment perspective which would say there are actual tangible improvements in access to justice for the marginalized and poor.

Her focus however is on individual agency of the women who suffer the violence and abuse and it is in a later article she acknowledges kinship and family ideology and practice that mediate notions of the individual actor (Lazarus-Black & McCall, 2006). In PNG, the various kinds of inhibitions or constraints that influence women making a formal complaint or seeking an order are inextricably tied to their natal family and kin and how taking action may negatively affect financial matters and reputations of this network and not just the individual (GHD, 2015; Médecins Sans Frontières, 2016). Our research shows that the support of these networks (family and friends) is equally critical in affecting the likelihood of the order resulting in some measure of protection. A subject that was only mentioned in passing by Lazarus-Black, is whether the law and the issuing of orders improved the safety of applicants, but which we asked about in our interviewees at various stages after they had applied and in some cases, secured an order.

Despite safety being the most important outcome from an order, determining whether an order has provided such protection in the short and longer term is no easy task. Resorting to evidence of recidivism on the part of the offender via reported and recorded breaches and further offenses is a very conservative and inappropriate measure, especially in a context like PNG where there is unreliable administrative record keeping. In our study, we relied on self-reported experiences of applicants at the time and immediate aftermath of their application, and for the second sample, the previous year during which the application was made. Our findings indicated the majority of applicants did feel safer and the majority would recommend applying for an IPO to a friend or family member, irrespective of whether there was a successful outcome to their application. It is worth noting that in the Solomon Islands, Ride and Soaki (2019) also found that where the police did issue a Police Safety Notice (under the domestic violence legislation) the majority (70%) of the recipients did feel safer. However, they found a wide variability in practice by police in how they dealt with complaints of domestic violence.

## Conclusion

On the basis of our research, we argue that the civil regime of protection orders is a crucial element of efforts to stop and reduce DFV. In PNG, the majority of women interviewed reported feeling safer, certainly in terms of short-term protection, although the prospects of longer term safety were less certain. Factors that contributed to likelihood of obtaining an order (and feeling safer) were having the support of significant others including family, and being a client of specialist FSV service, and the attitudes and values of local justice actors. The study indicated that many women did have access to their own money, and that even where many had taken refuge in safe houses, this did not necessarily lead to long-term separation. For the few who had experienced couple counseling, by the district court magistrate or by a non-justice actor, they were very positive about its impact, as for the applicants it meant they continued to live with their partners, who did change their behavior for the better. We heard how “empowered” women felt because of the state law that says they are entitled to live without violence. In other low-middle-income countries, analysis of demographic and household survey data suggests the domestic violence laws, even though not implemented well typically because of lack of a budget, do contribute to changes in attitudes and social norms. Although the survey results show a decline in the acceptability of domestic violence, the decline is mediated by gender, location, and class (Nguyen & Le, 2022).

In PNG, given the limitations of the formal criminal justice sector in dealing with FSV offenses (see Putt & Dinnen, 2020), the civil regime is more attuned and a better fit in the socio-cultural context, and alignment to the processes of the informal justice that occurs every day. Having acknowledged this, it is still important to recognize that it is fear and respect of the formal justice sector that often fosters compliance by the respondents. It is therefore worth continuing to invest in improving access to IPOs, that are more timely, and easily converted or issued separately as longer term POs. Structural factors, however, that weaken the formal justice system and result in uneven implementation of the FPA means that there is a cap on how much can be achieved. In practice, protection orders are not accessible to all, and we need to ask how access can be up-scaled. In addition, we have to be careful we do not exaggerate their impact. The very precarity of existence and the intermittent, partial, and episodic nature of state protection reinforces the social ethos of mutuality, with both negative and positive consequences. Donor funding and a cluster of specialized services and advocates, and sympathetic justice practitioners, draw upon the social ethos of mutuality to create localized “islands of hope.” However and importantly, there is not the capacity to expand, to spread access to these orders; except through perhaps the village courts and if the Act is amended. In the district courts and via the specialist Family and Sexual Violence Units, and other crucial health and support services such as the hospital Family Support Centres and the multiple yet isolated safe houses, there is not the capacity to deal with more and more order applications, let alone the enforcement of breaches or rapid responses to reported domestic violence crimes. It is possible that FPOs will remain a boutique option for some women in at best, most w-ban centers. Although there have been marked improvements in support and advocacy for victims through NGOs and FBOs, a more targeted approach by the PNG government and supported by donors is required to enable rapid issuing of interim orders by courts, to promote prompt and effective police responses to repo1ts of breaches, to amend provisions in the legislation to increase use in rural areas, and to promote understanding of the process among justice practitioners and networks of influential community leaders.

## Notes

1. Multiple terms such as intimate partner violence (IPV) and gender-based violence (GBV) are found in the literature and in research reports, but we use the term DFV, as it best describes how IPV or domestic violence is defined in the PNG Family Protection Act. However, in PNG it should be noted that family and sexual violence (FSV) is more commonly used term in the formal justice sector with for example, specialist police units called Family and Sexual Violence Units (FSVUs). In this article, we use DFV when referring to the general concept, and FSV when referring to PNG-specific services.

2. Beginning with a coalition of members of parliament and followed by a national summit, the Special Parliamentary Committee was established to inquire into issues related to GBV. The committee opened its proceedings in 2021, and its first report was released in September 2021. Its establishment and mode of operations, including public hearings and the calling of public servants and politicians, represents the first time GBV has been given a public political focus (see Kanan, 2021).

3. Out of the seven research locations, three did not have a local specialist FSV service to support survivors, and one did not have a local safe house.

4. The Act does allow for any person or a qualified legal practitioner, if written consent is given, or a police officer to apply for an order on behalf of a complainant, but except for one specialist FSV service, we did not find examples of this happening.

5. The rationale for and the composition of the two samples of applicants is explained in the next section.

6. Under the 2017 guidelines for the Act, it states that the court may direct the complainant or a member of the police force to serve documents or an order. We were told that frequently the complainant had to serve the summons or an order, largely because the police did not do it and not at the behest of the court. Our research underlined that this can be a particular time of risk for the complainant, especially given that respondents often acted angrily to the document or order.

7. The list of forms that relate to protection orders and are required to be completed at various junctures and by different parties is found in a table in our research report (see Putt & Kanan, 2021).

8. The Village Courts Regulations 1974 lists prescribed offenses, that include striking, threatening behavior, and offensive conduct. The Village Court Act 1989 s51 provides for penalties for failing to comply with preventive orders, which include fines and up to 6 months in prison.

9. The seven key conclusions were:

- FPOs are becoming more well known, but the processes are not well understood.
- Increasing awareness is important, but the system needs further funding and resources to respond to demand.
- Police have a critical role to play but do not always fulfill their role effectively.
- Specialist FSV services make a substantial difference by supporting and helping survivors both in times of crisis and over the longer term.
- Obtaining orders depends on access to district courts and committed magistrates.
- FPOs improve safety for most applicants; however, service of the order is a time of risk.
- Having family and church support can improve the effectiveness of FPOs and reduce the risks to DFV survivors

10. FemiliPNG is a special FSV service that supports survivors through a case management approach. An evaluation demonstrated its impressive track record in the assistance it provides to its clients (Putt, 2021).

11. Only in the National Capital District is there a separate family court, where many FPO applications are heard. In the other provinces, the district court will decide on sitting days and times for family court matters.

12. “Gardening” in PNG often refers to growing fruits and vegetables for household consumption or sale. “Marketing” refers to selling of that produce or other products at local markets.

13. Conditions that can be set under the Act, include three that relate to being apart or having no contact, but the first condition prohibits certain kinds of behavior. We found some courts would issue IPOs that employed a standard list of prohibitions related to not being violent etc.

14. That not all women who are issued orders are protected from violence and abuse is in keeping with research elsewhere (see Dowling et al., 2018) and the records of a specialist FSV service in PNG that adopts case management practices. Even though it has a good reputation for supporting victims, probably the best in the country for helping clients secure IPOs and PO, client data show that as many as one-fifth report abuse and violence post intake (Putt & Kanan, 2021).

## References

[bibr1-10778012251351912] Bott S. Morrison A. Ellsberg M. (2005). Preventing and responding to gender-based violence in middle and low-income countries: A global review and analysis [Policy Research Working Paper Series No. 3618]. The World Bank.

[bibr2-10778012251351912] Capshew T. F. McNeece C. A. (2000). Empirical studies of civil protection orders in intimate violence: A review of the literature. Crisis Intervention, 6(2), 151–167. 10.1080/10645130008951139

[bibr3-10778012251351912] Chan W. C. (2017). A review of civil protection orders in six jurisdictions. Statute Law Review, 38(1), 1–22. 10.1093/slr/hmv032

[bibr4-10778012251351912] Cokanasiga J. (2023). Criminal justice responses to domestic violence in Fiji. In Douglas H. Fitz-Gibbon K. Goodmark L. Walklate S. (Eds.), The criminalization of violence against women: Comparative perspectives (pp. 97–112). Oxford University Press.

[bibr5-10778012251351912] Colucci E. Hassan G. (2014). Prevention of domestic violence against women and children in low-income and middle-income countries. Current Opinion in Psychiatry, 27(5), 350–357. 10.1097/YCO.0000000000000088 25033276

[bibr6-10778012251351912] Conner D. H. (2015). Civil protection order duration: Proof, procedural issues and policy considerations. Temple Political and Civil Rights Law Review, 24(2), 343–374.

[bibr7-10778012251351912] Cordier R. Chung D. Wilkes-Gillan S. Speyer R. (2021). The effectiveness of protection orders in reducing recidivism in domestic violence: A systematic review and meta-analysis. Trauma, Violence and Abuse, 22(4), 804–828. 10.1177/1524838019882361 31658878

[bibr8-10778012251351912] Craig D. Porter D. (2018). Safety and security at the edges of the state: Local regulation in Papua New Guinea’s urban settlements. Justice and Development Working Paper Series. World Bank. https://documents1.worldbank.org/curated/en/184231530596208653/pdf/Safety-and-Security-at-the-Edges-of-the-State-local-regulation-in-Papua-New-Guinea-s-urban-settlements.pdf

[bibr9-10778012251351912] DeJong C. Burgess-Proctor A. (2006). A summary of personal protection order statutes in the United States. Violence Against Women, 12(1), 68–88. 10.1177/1077801205277720 16314662

[bibr10-10778012251351912] Demian M. A. (Ed.). (2023). Grassroots law in Papua New Guinea (monographs in anthropology). ANU Press.

[bibr11-10778012251351912] Department of Justice and Attorney General. (2017). Guidance notes for the Family Protection Act 2013. Government of Papua.

[bibr12-10778012251351912] Dinnen S. (2001). Law and order in a weak state: Crime and politics in Papua New Guinea, Pacific Islands Monograph Series 17. University of Hawai‘i Press.

[bibr13-10778012251351912] Dowling C. Morgan A. Hulme S. Manning M. Wong G. (2018, June). Protection orders for domestic violence: A systematic review, Trends and issues in crime and justice (Vol. 551). Australian Institute of Criminology.

[bibr14-10778012251351912] Ellsberg M. Arango D. Morton M. Gennari F. Kiplesund S. Conreras M. Watts C. (2015). Prevention of violence against women and girls: What does the evidence say? The Lancet, 385(Suppl. C), 1555–1566. 10.1016/S0140-6736(14)61703-7 25467575

[bibr15-10778012251351912] Ellsberg M. C. Heise L. (2005). Researching violence against women: A practical guide for researchers and activists. World Health Organization.

[bibr16-10778012251351912] Engle Merry S. (2006). Human rights and gender violence: Translating international law into local justice. University of Chicago Press.

[bibr17-10778012251351912] Frías S. M. (2024). Protection and access to justice of victims of gender-based violence in Mexico: An institutional critique. In Biholar R. Leslie D. L. (Eds.), Gender-based violence in the global south (pp. 143–163). Routledge.

[bibr18-10778012251351912] Gambetta V. Vanoli-Imperiale S. (2025). Women intimate partner violence revictimization during protection orders in Montevideo, Uruguay. Risk factors and policy implications. International Journal of Law, Crime and Justice, 80(Suppl. C), 100720. 10.1016/j.ijlcj.2024.100720

[bibr19-10778012251351912] Ganai T. (2017). Domestic violence cases at the Boroko District Court. Magisterial Service of Papua.

[bibr20-10778012251351912] GHD Pty Ltd. (2015). Evaluation of the RPNGC family and sexual violence units: Evaluation report. Australian Aid.

[bibr21-10778012251351912] Global Women’s Institute. (2022). Survivor-centred justice for gender-based violence in complex situations: Research report informed by case studies from Afghanistan, Honduras, Papua New Guinea, the Philippines, South Sudan, and Tunisia. International Development Law Organization and the Global Women’s Institute, George Washington University.

[bibr22-10778012251351912] Goddard M. (2009). Substantial justice: An anthropology of village courts in Papua New Guinea. Berghahn Books. 10.3167/9781845455613

[bibr23-10778012251351912] Goodale M. (2017). Anthropology and law: A critical introduction. New York University Press.

[bibr24-10778012251351912] Goodmark L. (2018). Decriminalizing domestic violence: A balanced policy approach to intimate partner violence (1st ed., Vol. 7). University of California Press.

[bibr25-10778012251351912] Heise L. (2011). What works to prevent partner violence: An evidence overview [Working Paper]. STRIVE Research Consortium, London School of Hygiene and Tropical Medicine.

[bibr26-10778012251351912] Holder R. (2018). Just interests: Victims, citizens and the potential for justice. Edward Elgar Publishing.

[bibr27-10778012251351912] Human Rights Watch. (2015). Bashed up: Family violence in Papua New Guinea. https://www.hrw.org/report/2015/11/04/bashed/family-violence-papua-new-guinea

[bibr28-10778012251351912] Jolly M. (2012). Introduction—Engendering violence in Papua New Guinea: Persons, power and perilous transformations. In Jolly M. Stewart C. Brewer C. (Eds.), Engendering violence in Papua New Guinea (pp. 1–46). ANU Press.

[bibr29-10778012251351912] Jolly M. Lee H. Lepani K. Naupa A. Rooney M. (2015, September). Falling through the net? Gender and social protection in the Pacific (Discussion Paper No. 6). UN Women.

[bibr30-10778012251351912] Kanan L. (2021, September 18). Spotlight on gender-based violence response in PNG, East Asia Policy Forum. https://eastasiaforum.org/2021/09/18/a-new-era-for-gender-based-violence-response-in-png/

[bibr31-10778012251351912] Kanan L. Putt J. (2021). Domestic violence and family law in Papua New Guinea (Research paper). Department of Pacific Affairs, Australian National University.

[bibr32-10778012251351912] Lazarus-Black M. (2001). Law and the pragmatics of inclusion: Governing domestic violence in Trinidad and Tobago. American Ethnologist, 28(92), 388–416. 10.1525/ae.2001.28.2.388

[bibr33-10778012251351912] Lazarus-Black M. McCall P. L. (2006). The politics of place: Practice, process and kinship in domestic violence courts. Human Organization, 65(2), 140–155. 10.17730/humo.65.2.5l4grc4qmk4rynuk

[bibr34-10778012251351912] Levitt P. Engle Merry S. (2009). Vernacularization on the ground: Local uses of global women's rights in Peru, China, India and the United States. Global Networks, 9(4), 441–461. 10.1111/j.1471-0374.2009.00263.x

[bibr35-10778012251351912] Mansour H. (2021). Covid-19's toll on Papua New Guinea. The Strategist, Australian Strategic Policy Institute, 14 October 2021.

[bibr36-10778012251351912] Maravuakula N. (2022). Regional overview of legislation relating to family protection orders. In Putt J. Kanan L. (Eds.), Family protection orders in the Pacific region (pp. 5–8). Department of Pacific Affairs, Australian National University.

[bibr37-10778012251351912] Médecins Sans Frontières. (2016, March). Return to abuser: Gaps in services and a failure to protect survivors of family and sexual violence in Papua New Guinea [Report]. https://www.msf.org/papua-new-guinea-new-msf-report-return-abuser-reveals-cycle-abuse-survivors-family-and-sexual

[bibr38-10778012251351912] Messing J. T. Bagwell-Gray M. E. Ward-Lasher A. Durfee A. (2021). ‘Not bullet proof’: The complex choice not to seek a civil protection order for intimate partner violence. International Review of Victimology, 27(2), 173–195. 10.1177/0269758021993338

[bibr39-10778012251351912] Myhill A. Johnson K. (2016). Police use of discretion in response to domestic violence. Criminology and Criminal Justice, 16(1), 3–20. 10.1177/1748895815590202

[bibr40-10778012251351912] Nguyen M. Le K. (2022). Can legislation reduce domestic violence in developing countries? Sustainability, 14(20), 13300. 10.3390/su142013300

[bibr41-10778012251351912] Office of Development Effectiveness. (2019). Ending violence against women and girls: Evaluating a decade of Australia’s development assistance. Department of Foreign Affairs and Trade, Commonwealth of Australia. https://www.dfat.gov.au/sites/default/files/evawg-final-report-nov-19.pdf

[bibr42-10778012251351912] Ohlin L. E. Remington F. J. (1993). Discretion in criminal justice: The tension between individualization and uniformity. SUNY Series in New Directions in Criminal Justice 1 2 Studies. State University of New York Press.

[bibr43-10778012251351912] Putt J. (2021). Helping family and sexual violence survivors in Papua New Guinea: Evaluation of Femili PNG, Lae Operations 2014–2020. Department of Pacific Affairs, Australian National University.

[bibr44-10778012251351912] Putt J. Dinnen S. (2020). Reporting, investigating and prosecuting family and sexual violence offences in Papua New Guinea. Department of Pacific Affairs, Australian National University.

[bibr45-10778012251351912] Putt J. Kanan L. (2021). Family protection orders in Papua New Guinea—Main report. Department of Pacific Affairs, Australian National University.

[bibr46-10778012251351912] Putt J. Kanan L. (Eds.). (2022). Family protection orders in the Pacific region. Department of Pacific Affairs, Australian National University.

[bibr47-10778012251351912] Putt J. Phillips T. Thomas C. Kanan L. (2019). The use and efficacy of family protection orders as a key response to domestic and family violence: A pilot study in Lae. Department of Pacific Affairs, Australian National University.

[bibr48-10778012251351912] Richards T. N. Tudor A. Gover A. R. (2018). An updated assessment of personal protective order statutes in the United States: Have statutes become more progressive in the past decade? Violence Against Women, 24(7), 816–842. 10.1177/1077801217722237 29332498

[bibr49-10778012251351912] Ride A. Soaki P. (2019). Women’s experiences of family violence services in Solomon Islands. Australian Aid/Solomon Islands Government.

[bibr50-10778012251351912] Sustineo Pty Ltd. (2023). Support for the law and justice sector’s response to family and sexual violence study 2016–2021: Final Report [Report]. Justice Services and Stability for Development Program, Sustineo Pty Ltd.

[bibr51-10778012251351912] Turhan Z. Genç E. Başer Baykal N. (2025). Lived experiences of protection orders among women survivors of domestic violence. Women & Criminal Justice, 35(3), 191–204 . 10.1080/08974454.2023.2228769

[bibr52-10778012251351912] Tyedmers H. (2024). Troubling kastom: Women, violence and justice in Vanuatu [Unpublished doctoral dissertation]. Australian National University. https://hdl.handle.net/1885/733724848

[bibr53-10778012251351912] UN Women. (2023). Facts and figures: Ending violence against women. https://www.unwomen.org/en/what-we-do/ending-violence-against-women/facts-and-figures#83921

[bibr54-10778012251351912] Wiltshire C. (2016). Public expenditure, decentralisation and service delivery in Papua New Guinea: Tracking budgets to health clinics [Unpublished doctoral dissertation]. Australian National University. https://openresearch-repository.anu.edu.au/handle/1885/119220

